# 6.H. Workshop: Towards improved performance measurement and universal health coverage in oral health care

**DOI:** 10.1093/eurpub/ckac129.370

**Published:** 2022-10-25

**Authors:** Organised by: European Observatory on Health Systems and Policies, Technische Universität Berlin, Radboud University, New York University, Chair persons: Ewout van Ginneken (Germany), Dimitra Panteli (Belgium)

## Abstract

Oral diseases are increasingly recognized as one of the most prevalent conditions in Europe, affecting nearly half of the European population. They are the third most expensive diseases to treat in the EU and dental expenditures are set to rise substantially. Within EU countries, oral health policy and financing are most often separated from the general health system and vary considerably between countries with different public and private organisation structures providing oral health services, different levels of out-of-pocket payments for patients and a variety of financing models. Oral diseases are a main driver of unmet need and financial hardship in the EU and particularly affect poorer and marginalized groups. However, current provider payment systems prioritize expensive invasive and technology-based methods which drives public expenditure while disserving the healthcare system's central objective of better health outcomes and reducing waste. Moreover, broad and effective oral health promotion policy that spans various care settings and areas of life is not sufficiently implemented. Oral health care systems thus face complex challenges regarding efficiency, quality and integration of care which requires good evidence to steer oral health policy. However, current health information systems only collect few indicators on oral health and oral health care. There is thus need for an improved evidence base for more meaningful assessments and comparisons of oral health systems performance which would allow better informed policy decisions and enable more targeted and effective oral health interventions. In May 2021 the World Health Assembly approved an historic resolution on oral health which urges Member States to include oral health in universal health coverage (UHC) benefit packages and to shift from the traditional curative approach towards a preventive approach. Oral health and the urgent needs for improving the financing of oral health systems have thereby received increased policy attention also in Europe. In this workshop evidence from a cross-country comparison of oral health financing, access and provision in Europe will be presented and the lack of supportive information systems for oral health system performance assessment will be highlighted. Recent research activities will be discussed such as the Horizon Europe project DELIVER (DELiberative ImproVEment of oRal care quality) which aims to create a synergistic problem-solving ecosystem to improve oral health systems. Debates in oral health research and policy in Europe are influenced by global developments such as the WHO resolution. To this end, the recent call for urgent system reform and policy action to address the burden of oral diseases will be set in context and discussed.

**Key messages:**

• Oral care systems are still dominated by a traditional curative and technology-driven approach which fail to sufficiently encourage prevention and support holistic and integrated oral health care.

• Lack of harmonized data reporting on oral health and oral health system indicators impedes oral care systems performance monitoring and evidence-informed policy-making.

